# Observation of Coherent Spin Waves in a Three-Dimensional Artificial Spin Ice Structure

**DOI:** 10.1021/acs.nanolett.1c00650

**Published:** 2021-05-28

**Authors:** Sourav Sahoo, Andrew May, Arjen van Den Berg, Amrit Kumar Mondal, Sam Ladak, Anjan Barman

**Affiliations:** † Department of Condensed Matter Physics and Material Sciences, S. N. Bose National Centre for Basic Sciences, Block JD, Sector III, Salt Lake, Kolkata 700 106, India; ‡ School of Physics and Astronomy, Cardiff University, Cardiff CF24 3AA, U.K.

**Keywords:** 3D nanomagnetism, 3D lithography, 3D artificial spin ice structure, spin dynamics, spin waves, Brillouin light scattering

## Abstract

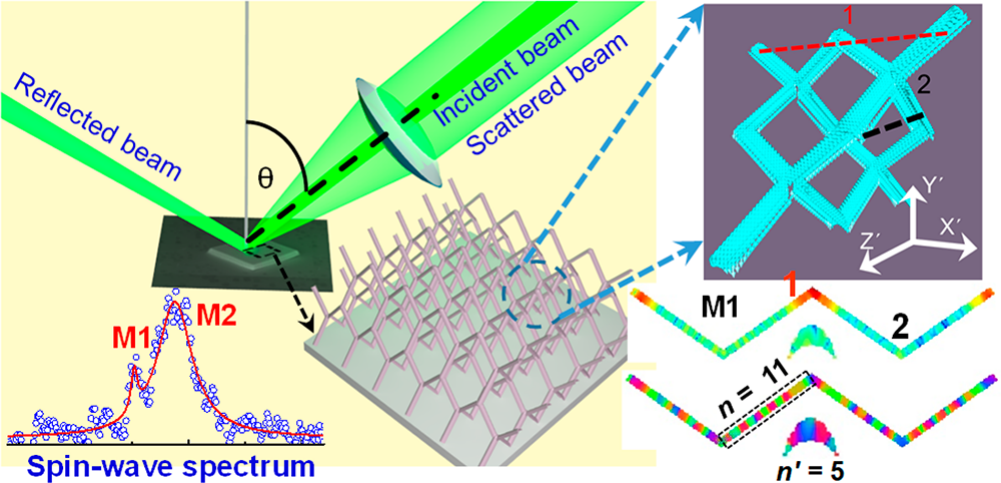

Harnessing high-frequency spin dynamics in three-dimensional (3D) nanostructures may lead to paradigm-shifting, next-generation devices including high density spintronics and neuromorphic systems. Despite remarkable progress in fabrication, the measurement and interpretation of spin dynamics in complex 3D structures remain exceptionally challenging. Here, we take a first step and measure coherent spin waves within a 3D artificial spin ice (ASI) structure using Brillouin light scattering. The 3D-ASI was fabricated by using a combination of two-photon lithography and thermal evaporation. Two spin-wave modes were observed in the experiment whose frequencies showed nearly monotonic variation with the applied field strength. Numerical simulations qualitatively reproduced the observed modes. The simulated mode profiles revealed the collective nature of the modes extending throughout the complex network of nanowires while showing spatial quantization with varying mode quantization numbers. The study shows a well-defined means to explore high-frequency spin dynamics in complex 3D spintronic and magnonic structures.

Patterned magnetic nanostructures have been studied a great deal during the last few decades due to their interesting spin configurations^1−3^ and their potential applications in energy-efficient miniaturized spintronics as well as magnonic devices^4−6^ where spin waves (SWs) may act as information carriers. A large volume of work has been done on one-dimensional (1D) and two-dimensional (2D) magnonic crystals (MCs) of different form and geometry.^2,7−10^ These include magnetic dot arrays,^11^ antidot arrays,^12^ bicomponent magnonic crystal (BMC),^13^ nanowires,^14^ and nanostripes.^15^ During the past few years, three-dimensional (3D) nanomagnetism has emerged as a fascinating research field demonstrating novel physical phenomena such as curvature-induced anisotropy,^16,17^ frustration in 3D artificial spin ice (ASI) systems,^18,19^ 3D MCs,^17,20^ and noncollinear spin textures such as twisted skyrmions,^21^ magnetic singularities, e.g., Bloch points,^22,23^ hopfions,^24^ and vortex domain walls.^25^ On the other hand, 3D magnetic nanostructures have the potential for future applications in magnetic sensors,^26^ neuromorphic computing,^27^ ultradense data storage devices,^28,29^ and 2.5D spintronic devices.^30^ The main barriers to exploration of 3D magnetic nanostructures^30−33^ have been their nontrivial fabrication and characterization techniques. The combination of 3D patterning techniques such as focused electron beam-ion deposition (FEBID),^34^ two-photon lithography (TPL)^35−38^ with sputtered deposition,^39^ electrodeposition,^40^ and thermal evaporation^19,41^ has emerged as powerful techniques to fabricate 3D complex magnetic nanostructures for investigation of novel phenomena and development of future magnetic devices. Recently, high-quality, free-standing tetrapod structures have been made from Co nanowires, by utilizing TPL and electrodeposition.^40^ The SW dynamics from the junction of a tetrapod structure was experimentally measured using a time-resolved Kerr microscope.^42^ However, the large separation between the tetrapods did not allow the study of coherent magnons in this system. The study of SW dynamics within interconnected 3D magnetic nanostructures is important to first of all build an elementary understanding of SW mode behaviors within such complex systems and subsequently to develop future devices which allow the propagation of SWs to be controlled in complex 3D circuits. Such structures hold the promise to study coherent magnon states in 3D MCs due to Bragg scattering in all three spatial directions, as well as investigation of anisotropic magnon minibands and Brillouin zone boundaries along high-symmetry directions. Theoretical studies of SW dynamics in a prototype of 3D interconnected magnetic nanostructures^20,43^ have been reported recently. Finally, the realization of 3D magnetic nanostructures in complex frustrated geometries, such as a 3D-ASI, provides access to a huge number of near degenerate states. Here, previous work has shown the possibility of quasi-3D-ASI systems^44,45^ through use of multiple lithography steps. These novel systems show a degenerate ice rule and have demonstrated the square ice model, though such systems are currently limited to two layers. In contrast, harnessing self-assembly techniques and electrodepostion, true 3D geometries have been obtained, which have an inverse opal geometry.^18,46^ Two-photon lithography and deposition have recently emerged as a powerful means to produce 3D-ASI systems, taking a diamond-bond lattice (DBL) geometry.^19,47^ A distinct advantage of these systems is the direct-write capability which allows the 3D nanoscale geometry to be written by design. Furthermore, direct magnetic imaging of such structures has now shown the possibility of realizing the full suite of vertex types, as seen in 2D-ASI systems, providing a platform to explore SW dynamics in 3D-ASI for construction of reconfigurable magnonic devices.^48^

Here, we report upon the experimental measurement of SW modes in a 3D-ASI composed of interconnected nanowires arranged in DBL structure using conventional Brillouin light scattering (BLS). The 3D-ASI was fabricated by using a combination of TPL and thermal evaporation. Two clear SW modes were observed in the BLS spectra, each of which showed a systematic variation with the applied magnetic field. These experimental results have been understood in the context of 3D micromagnetic simulations, which show the observed modes can be reproduced in the simulation. The simulated mode profiles revealed complex quantized characters with its power distributed over the entire structure.

A 3D array of interconnected nanowires of DBL (3D-DBL) was fabricated by using a three-step process. In the first step, a 3D-DBL structure (50 × 50 × 10 μm^3^) was fabricated upon glass using TPL and subsequent development. In the second step, a layer of gold (30 nm) was deposited upon the sidewalls of the scaffold nanowires. This was achieved by carrying out four separate Au depositions, whereby the sample was mounted at a 30° tilt and the in-plane angle was rotated by 90° for each deposition. The addition of 4 × 30 nm Au depositions upon the polymer sidewalls allows efficient dissipation of heat for long optical experiments (see Section S1 of the Supporting Information). Finally, a 50 nm-thick Ni_81_Fe_19_ (Permalloy; Py hereafter) was deposited with the substrate in a flat, zero-tilt position. The deposition of Py on the curved surfaces leads to the formation of nanowires with a crescent-shaped cross-section.^19,47^ Overall, the process yields a DBL of Py which is continuous for four layers, in the *y*-direction, corresponding to a unit cell in thickness.^19^

Scanning electron micrographs of the full 3D array are shown in Figure 1(a), and a magnified view (inset of Figure 1(a)) shows a constituent tetrapod element of the interconnected nanowire structure. The four sublattice layers are annotated in Figure 1(b). The individual nanowire length is approximately 1000 nm, and its width is approximately 260 nm. A deviation up to ±10 nm in width and ±25 nm in length of the nanowires was observed. Magnetic force microscopy showed similar contrast to previous samples indicating single domain wires (see Section S1 of the Supporting Information). More details of fabrication and characterization of the samples can be found elsewhere.^19^Figure 1(c) shows the magnetic hysteresis loop (Kerr rotation (θ_K_) vs magnetic field (*H*)) of the 3D-ASI sample measured using the static magneto-optic Kerr effect (MOKE) technique, which gives the saturation field and coercive field as ∼125 Oe and ∼100 Oe, respectively. In the SW measurements, the bias magnetic field was always kept well above the saturation field, ensuring a single-domain state of the sample.

**Figure 1:**
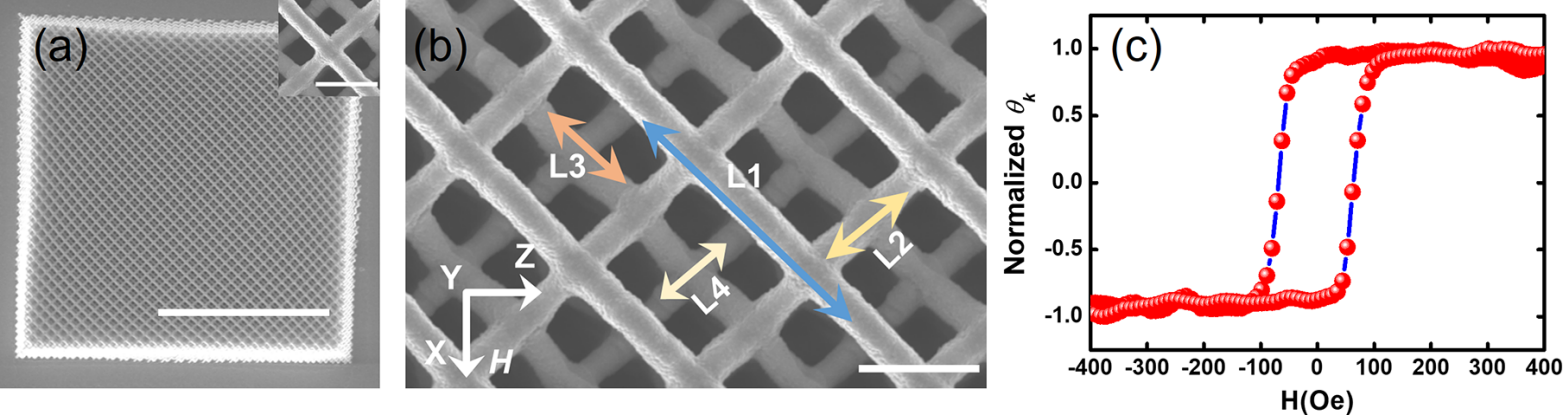
(a) Scanning electron micrograph showing a top view of the full array of 3D-ASI with size 50 × 50 × 10 μm^3^. The scale bar is 25 μm. A constituent tetrapod element is shown in the inset by capturing a zoomed view of a 3D array where the scale bar is 1 μm. (b) A magnified view of the interconnected nanowires in the lattice is shown. Four sublattice layers are highlighted. Scale bar in (b) is 2 μm. (c) Magnetic hysteresis loop of the 3D-ASI sample measured using a static magneto-optical Kerr effect. Applied magnetic field (*H*) geometry is shown in (b).

The SW dynamics of the 3D array were measured by using conventional BLS.^49^ BLS is a popular tool to measure SW dynamics of magnetic thin films and patterned nanostructures. It is a noncontact and thus noninvasive tool to measure thermally excited SWs at room temperature without any external excitation and under ambient conditions. This technique relies upon inelastic scattering of light from the sample. The mechanism of inelastic scattering can be quantum mechanically described as a photon–magnon collision, where the creation (Stokes process) and annihilation (anti-Stokes process) of a magnon of wave vector (*k*) and angular frequency (ω) are detected. A continuous wave of monochromatic laser light (wavelength λ = 532 nm, power = 60 mW) was focused on the sample (Figure 2(a)) to a spot size of around 40 μm, which is close to the lateral dimensions of the sample. As a result, the SWs were measured from almost the entire sample volume. The cross-polarization between the inelastically backscattered beam and incident beam was exploited to suppress the phonon contribution. A Sandercock-type six-pass tandem Fabry–Perot interferometer was used to analyze the frequencies of the scattered beam, in order to extract the SW frequencies. In our experiment, we applied a bias magnetic field (*H*) parallel to the substrate plane as shown in the inset of Figure 2(a), along a principal axis (*x*-direction) of the lattice. A high magnetic field was first applied to completely saturate the sample magnetization, which was then gradually decreased to each bias field value for the BLS measurement. A schematic of the experimental geometry is shown in Figure 2(a).

**Figure 2:**
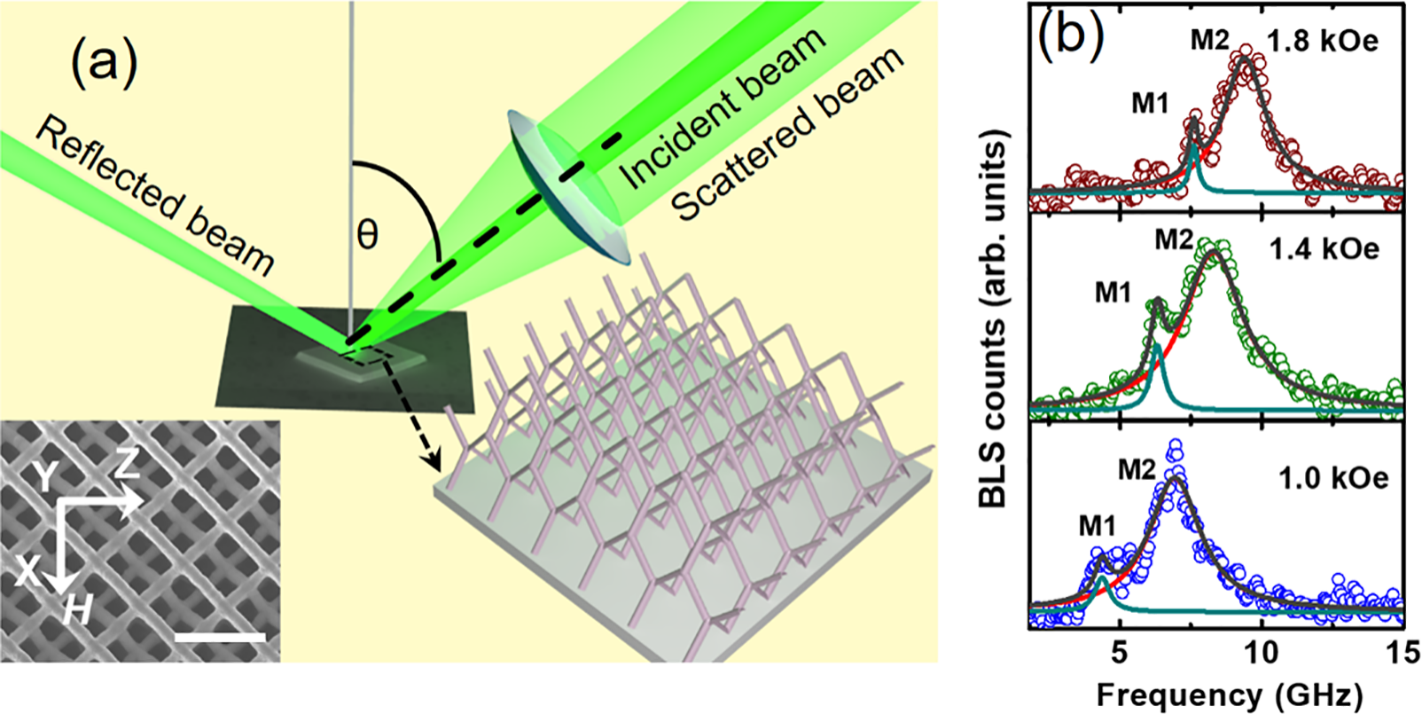
(a) Schematic of BLS measurement geometry. The measurement was performed at θ = 0°. The applied field (*H*) direction is shown in the left inset, and the scale bar is 2 μm. The 3D-ASI network is presented by a schematic on the right side of the image. (b) BLS spectra for three different magnetic field values are shown. Open circles present the experimental data points. Here, cyan and red color solid lines present the fitting of individual peaks, and the gray color solid line presents the results of the multipeak fitting.

In order to study the SW frequency variation with *H*, the BLS spectra were measured for the *k* ≈ 0 wavevector in the Damon–Eschbach (DE) geometry corresponding to scattering of photons by a surface magnon, for different *H* values in 0.6 ≤ *H* ≤ 2.0 kOe. Here the momentum will be conserved only in the plane of the sample surface. The *k* ≈ 0 geometry will allow us to measure the extended and localized SWs over the entire 3D network of connected nanowires but not the SW frequency vs wave vector dispersion. Some example BLS spectra from the 3D-ASI are shown in Figure 2(b). Two intense SW modes are observed in the spectra which are named as M1 and M2. The lower frequency peak (M1) becomes more prominent at larger values of *H*. The higher frequency mode (M2) is quite broad apparently due to unresolved modes and/or inhomogeneous line broadening due to defects and inhomogeneous spin textures in the real sample. The BLS spectra were fitted with two-peak Lorentzian functions to extract the SW frequency values. The bias magnetic field variation of SW frequencies is plotted in Figure 3(a) with combined error bars originating from theoretical fits and the frequency resolution of the BLS setup. The latter is given by the instrumental line width, which is ∼0.3 GHz, determined from the elastic peak as shown at the inset of Figure 3(a). The SW mode frequency increases nearly monotonically with increasing field values, suggesting purely magnetic origin of the modes. Despite the complicated structure of the 3D-ASI and the corresponding demagnetizing factors, we have fitted the most intense mode with the Kittel formula, which resulted in a good fit with effective demagnetizing factors at three different axes as presented in Section S2 of the Supporting Information.

**Figure 3:**
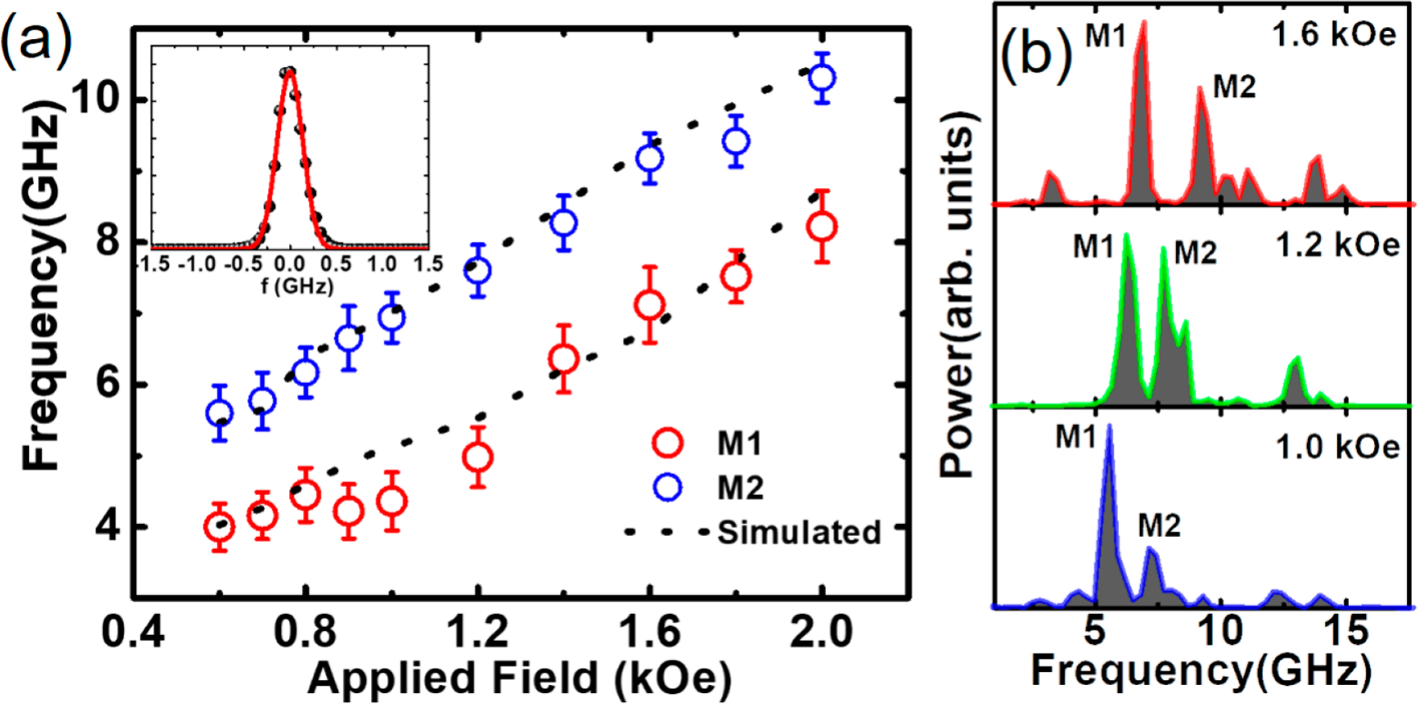
(a) Spin-wave frequencies of mode 1 (M1) and mode 2 (M2) are plotted as a function of applied magnetic field. Here, symbols present the experimentally measured data points. The elastic peak fitted with a Gaussian function is shown at the inset. (b) Simulated spin-wave spectra for three intermediate field values are shown. The dotted lines in (a) present the simulated results.

To obtain deeper insight into the behavior of the observed SW modes, we have numerically simulated the SW dynamics in the 3D-ASI system using the GPU-based MuMax3 software.^50^ A schematic of the diamond-lattice unit cell^51^ is shown in Figure 4(a) for clarity of understanding of the 3D-ASI structure, where the atoms are nonexistent and only the bonds are present. A typical simulated static spin configuration of the 3D-ASI structure is shown in Figure 4(b), which consists of four tetrapod elements, highlighted by different colors. The 3D-ASI structure has been designed to match the geometry of a DBL, as shown in Figure 4(a). In order to mimic the experimental sample volume, we considered a unit cell of the 3D-ASI in the simulation and applied a 2D periodic boundary condition in the *x*–*z* plane, while along the *y*-direction the simulated structure contains four layers similar to the experimental sample. The simulated unit cell of the 3D-ASI consists of crescent-shaped nanowires (CSNs) with dimensions similar to the experimental sample. The sample was divided into cuboidal cells of size 5 × 5 × 5 nm^3^. The cell size is taken below the exchange length of Py (≈5.2 nm). Further test simulations with lower cell size made no significant qualitative changes in the peaks of interest in the simulated SW spectra. The material parameters used in the simulation are gyromagnetic ratio γ = 17.6 MHz/Oe, saturation magnetization *M*_s_ = 860 emu/cc, anisotropy field *H*_K_ = 0, and exchange stiffness constant *A*_ex_ = 13 × 10^–7^ erg/cm for Py.^52^ The equilibrium magnetic states were obtained by relaxing the sample under study at a fixed bias magnetic field along the *x*-direction as defined in Figure 4(c). The magnetization configuration of the equilibrium state (m_*x*_ component) at *H* = 1.6 kOe is shown in Figure 4(c) which shows a saturated state along the *x*-direction with very small demagnetized regions. The magnetization configurations (**m**_*x*_ component) at few other bias magnetic fields are presented in Figure S4 of Section S3 of the Supporting Information. The equilibrium state magnetization configurations of two other components (**m**_*y*_ and **m**_*z*_) corresponding to Figure 4(c) are shown in Figure S5 of Section S3 of the Supporting Information. For simulation of the SW dynamics, a square-shaped pulsed magnetic field with peak amplitude of 20 Oe along the *y*-direction with rise and fall time of both 10 ps and duration of 20 ps was applied to the equilibrium magnetic state using a Gilbert damping parameter α = 0.008. The SW spectra were calculated by taking fast Fourier transformation (FFT) of the simulated time-domain magnetization (**m**_*y*_ component). Some typical simulated SW spectra are shown in Figure 3(b). The simulated spectra show additional peaks which were not resolved due to either the line broadening or insufficient sensitivity due to their smaller power in the experiment. The peaks adjacent to M2 observed in simulation may have contributed to broadening of M2 in the BLS spectra as mentioned before. Nevertheless, both the experimental peaks could be identified in the simulation, and the variation of their frequencies with bias magnetic field is plotted as dotted lines in Figure 3(a). A qualitative agreement between the experimental and simulated bias-field variation of SW frequencies is found, although the frequency of M1 from BLS shows larger scatter beyond the error bars in the lower bias field regime presumably due to its reduced intensity and increased noise in these fields, which pose additional uncertainty in determining its peak frequency. The collective nature of the SW modes has been reconfirmed by test simulations of SW spectra of a single nanowire leg, one tetrapod element, and a 3D-ASI structure, which clearly show gradual evolution from SW modes localized in its constituent elements to a 3D-ASI lattice (Section S4 of the Supporting Information). To understand the spatial nature of the coherent SW modes in the 3D-ASI, we have further analyzed the simulated data from MuMax3 using a home-built post processing code.^53^ The scheme of the post processing is briefly discussed in Section S5 of the Supporting Information. The mode profiles were calculated by taking a slice of 3D-ASI structure along red and black dashed lines, parallel to different planes at positions 1 and 2, respectively, as shown in Figure 5(a). The coordinate system *x*′*y*′*z*′ in Figure 5(a) is defined such that the projections of top and bottom layers on the *x*′–*z*′ plane become parallel to the *x*′ and *z*′ axis, respectively. The simulated powermaps of the experimentally observed SW modes are shown in Figure 5(b), 5(c), and 5(d) for *H* = 1.6 kOe. The powermaps of additional modes present only in the simulated spectra are presented in the Section S6 of the Supporting Information. The calculated powermaps along the red dashed line parallel to the *x*′–*y*′ plane at position 1 are shown in Figure 5(b). It captures the lateral view of the top layer and one of the bottom layer nanowire’s cross-sectional view. The lateral view reveals that the power of M1 is localized at the junction of each bipod, but the power of M2 is extended throughout the 3D-ASI nanowire network as shown in Figure 5(b). The phase of the modes M1 and M2 reveal quantized nature with different quantization numbers *n* = 11 for M1 and *n* = 5 for M2 as shown by dashed lines in Figure 5(b). The cross-sectional view of CSN in the bottom layer reveals M1 to have quantized nature at the CSN cross-section (*n′* = 5) with low power, whereas M2 also possesses quantized nature (*n′* = 2) with reasonably high power as shown in Figure 5(b). To explore the mode profiles further, we took another slice along the black dashed line parallel to the *y*′–*z*′ plane at position 2 (Figure 5(c)). Here, power is distributed over the entire nanowire network for both modes. M1 shows quantized nature with *n* = 9, while M2 also shows quantized nature with *n* = 7 in both the connecting nanowires. The mode profile behavior at the CSN cross-section was also analyzed by taking a slice along the red dashed line parallel to the *x*′–*z*′ plane at position 1, as shown in Figure 5(d). It captures mode profiles near the junction at position 1 and crescent-shaped cross-sections of two adjacent nanowires. Here, M1 forms a quantized mode at the CSN cross-section of a junction with *n′* = 3, while M2 also forms a quantized mode with *n′* = 2. The overall power profile suggests that M1 is primarily localized at the nanowire junctions, while M2 is extended over the nanowire networks. The phase profiles reveal the 3D nature of these modes with different quantization numbers along the lateral direction of nanowires and at the cross-sections. We have applied the magnetic field along one principal axis (along the *x*-axis, Figure 4(a)) which is an in-plane symmetry axis for the 3D-ASI structure. However, the crescent cross-section of the nanowires and the 3D geometry of ASI may create a 3D varying magnetic potential within this nanostructure, leading to the quantized nature for both SW modes with different quantization number.

**Figure 4:**
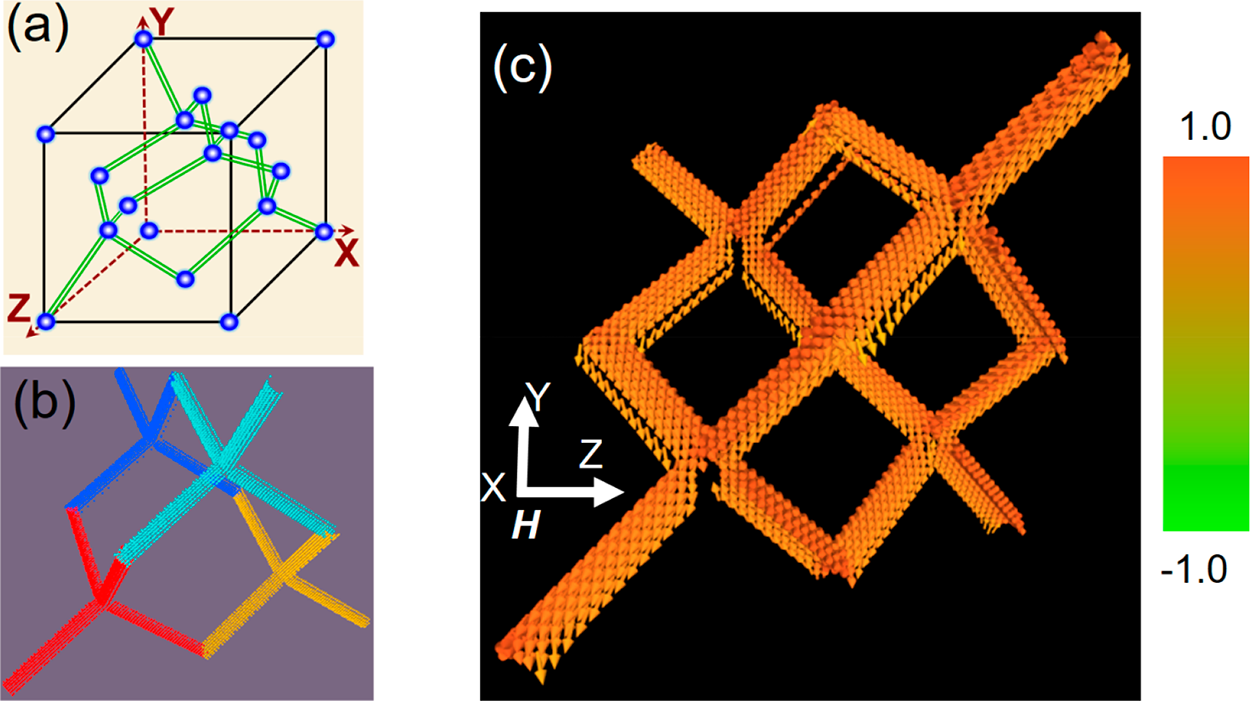
(a) Schematic of a diamond-lattice unit cell. Here, the green colored double lines present the bond position, and blue spheres present the atom position. The dashed lines define the axes. (b) A representative simulated unit cell of 3D-ASI is shown. One unit cell consists of four tetrapod elements, and each of them are highlighted by a different color for better visualization. A one-to-one correspondence between the diamond bond lattice and 3D-ASI (omitting the atoms) could be found from (a) and (b). (c) Magnetization configuration of the equilibrium state (**m**_*x*_ component) at *H* = 1.6 kOe (along *x*-direction; in-plane) is shown. The color bar for **m**_*x*_ is shown next to the image.

**Figure 5:**
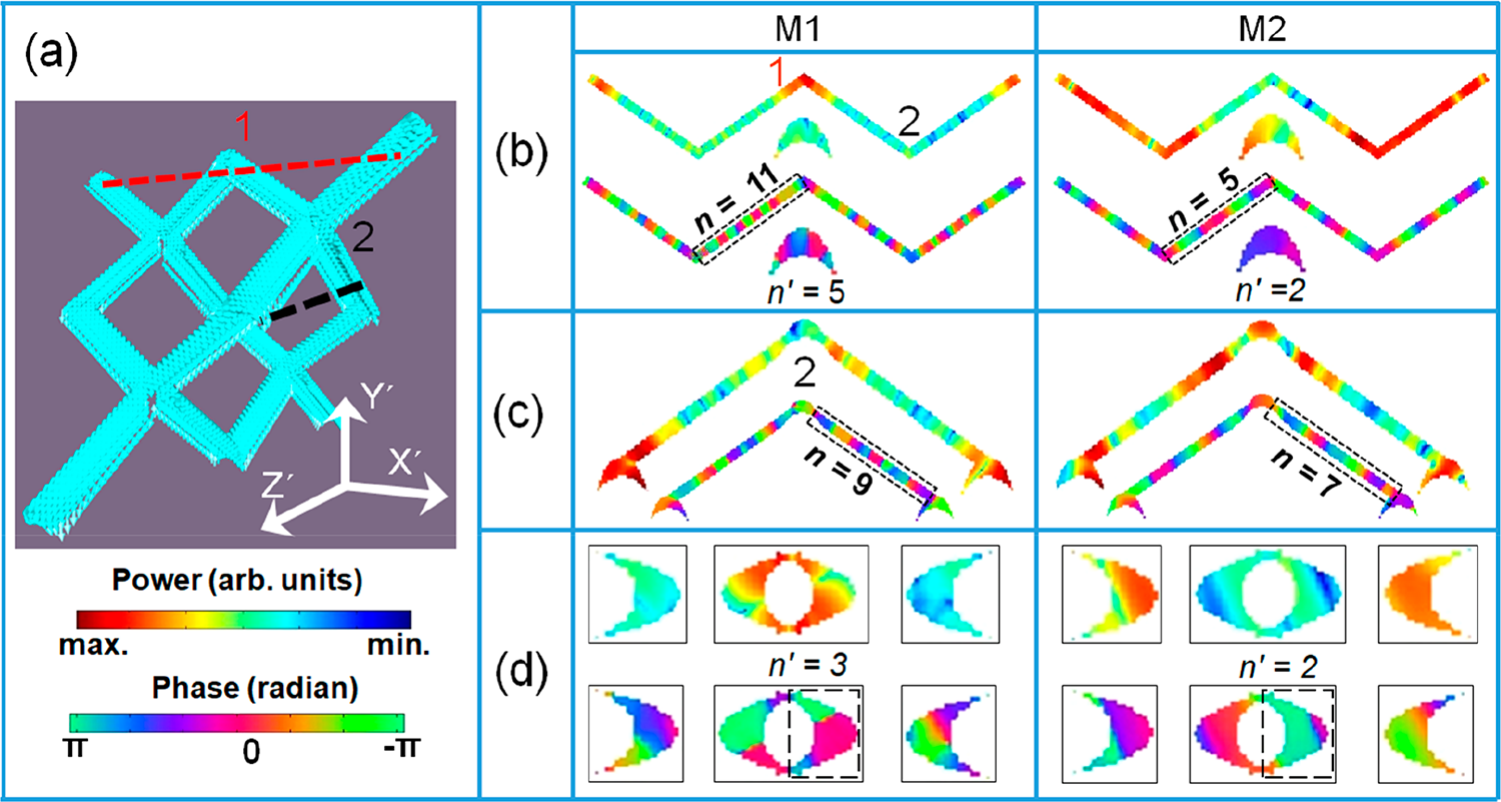
(a) Representative static magnetization profile (**m**_*x*_ component) along with the coordinate system. The red and black dashed lines show the slice taking positions to extract mode profiles parallel to different planes at positions 1 and 2. Spin-wave mode profiles are calculated at different points of the 3D-ASI structure at *H* = 1.6 kOe by taking a slice along: (b) the *x*′–*y*′ plane at point 1, (c) the *y*′–*z′* plane at point 2, and (d) the *x*′–*z*′ plane at point 1. The power profiles are shown in the upper panels and the corresponding phase profiles in the lower panels. The color bars for power and phase are shown at the bottom left corner.

In summary, we have exploited a novel method to fabricate a complex 3D-ASI structure of interconnected nanowires arranged in a diamond-bond-like lattice using TPL and thermal evaporation. We have studied thermal magnons in this 3D-ASI using BLS spectroscopy which revealed two clear collective SW modes. The SW mode frequencies show good stability and nearly monotonic variation over a broad range of bias magnetic fields. We have performed 3D micromagnetic simulations to reproduce the SW spectra and obtained insights into the spatial nature of the SW modes in this 3D-ASI. The SW modes exhibit different spatial characters from localized to extended nature having different mode quantization numbers. Some additional modes in the simulation were either not resolved or detected in the experiment, which called for a more precise experiment to detect those. On the other hand, the fabrication of even higher quality samples extended equally in all three dimensions will be helpful to understand the SW propagation along all high-symmetry axes in these structures. The collective SW modes in this 3D-ASI system are starkly different from an isolated 3D nanostructure, as in the former the coherent SW can extend over all three dimensions along a complex 3D network, promising a very rich magnonic band structure with greater flexibility as opposed to the formation of standing SWs in an isolated 3D nanostructure. Besides, these SWs can be highly reconfigurable due to the possibility of attaining a variety of complex magnetic microstates by varying external magnetic field.^47^ To this end, optical exploration of SW modes in this interconnected 3D structure will open the pathway for exciting new possibilities of 3D magnonics for ultrahigh density on-chip communication and processing devices.

## Supporting Information Available

S1. Sample fabrication for prolonged optical exposure. S2. Bias field-dependent spin-wave frequency. S3. Static magnetization configuration of the sample. S4. Collective nature of spin-wave modes. S5. Spin-wave mode profile calculation procedure. S6. Calculated spin-wave mode profiles of additional modes observed only in the simulation. S7. Spin-wave mode profiles of adjacent frequencies (PDF)
