# Supplementary material for: Observation of Coherent Spin Waves in a Three-Dimensional Artificial Spin Ice Structure
Source: Nano Lett. 2021 May 28;21(11):4629–35. doi: 10.1021/acs.nanolett.1c00650 (PMC8289297; doi:10.1021/acs.nanolett.1c00650)
Supplement: Supplementary file 1 — nl1c00650_si_001.pdf [file nl1c00650_si_001.pdf]

Supplementary Information

Observation of Coherent Spin Waves in a Three-Dimensional Artificial Spin Ice Structure

Sourav Sahoo[1], Andrew May[2], Arjen van Den Berg[2], Amrit Kumar Mondal[1], Sam Ladak[2] and Anjan Barman[1]*

1. Department of Condensed Matter Physics and Material Sciences, S. N. Bose National Centre for Basic Sciences, Block JD, Sector III, Salt Lake, Kolkata 700 106, India

2. School of Physics and Astronomy, Cardiff University, Cardiff CF24 3AA, UK

*abarman@bose.res.in

S1. Sample Fabrication for Prolonged Optical Exposure

The fabrication of these novel 3D-ASI systems was carefully optimised to ensure consistency with previous results, single domain behaviour and robustness with respect to prolonged optical exposure. Initially, simple optical experiments were performed in Cardiff University upon samples which consisted of the underlying polymer diamond lattice and a single layer of $Ni_{81}Fe_{19}$ (Py). Here samples were illuminated for approximately 4 hours, and subsequently inspected. Figure S1 shows scanning electron microscope (SEM) images of the lattice after exposure showing obvious damage.

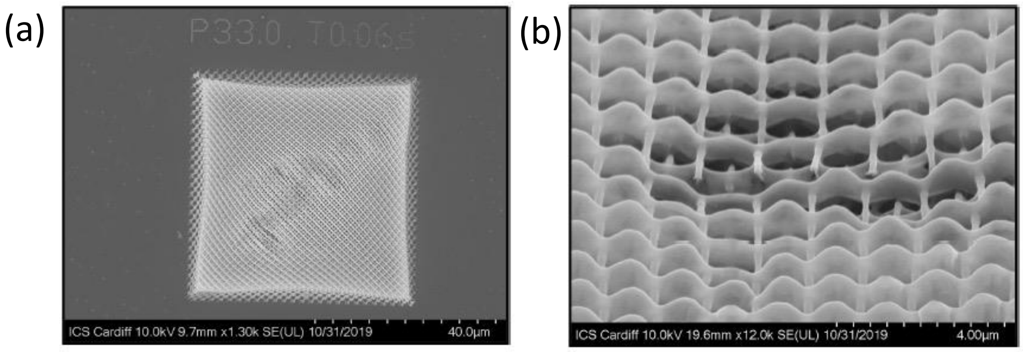

Figure S1: Scanning electron microscope image of (a) an entire 3D-ASI sample and (b) selected region of the sample after exposure to optical excitation (6 mW, spot size ~ 20 μm) for 4 hours.

Therefore, a process was developed whereby four subsequent Au depositions were performed with the sample mounted at a 30-degree tilt and the in-plane angle was rotated by 90-degrees for each deposition. This allowed more efficient dissipation of heat across the lattice and to the substrate. A further 50-nm-thick $Ni_{81}Fe_{19}$ layer was then deposited with the substrate in a flat, zero-tilt position. Prolonged optical exposure for >4 hours then yielded no damage. This was also checked via SEM after Brillouin light scattering (BLS) measurements. To ensure the underlying nanowires within the new system were single domain, samples were subject to atomic force microscopy (AFM) and magnetic force microscopy (MFM). Figure S2 shows the AFM and MFM of the as-deposited samples. The AFM in Fig. S2a shows clear measurement of L1 and L2 sub-lattices. Figure S2b shows clear magnetic contrast upon the L1 sub-lattice. The majority of bipods upon the upper surface appear to be in a 1-in/1-out state with contrast resembling our previous studies [1,2] indicating single domain behaviour.

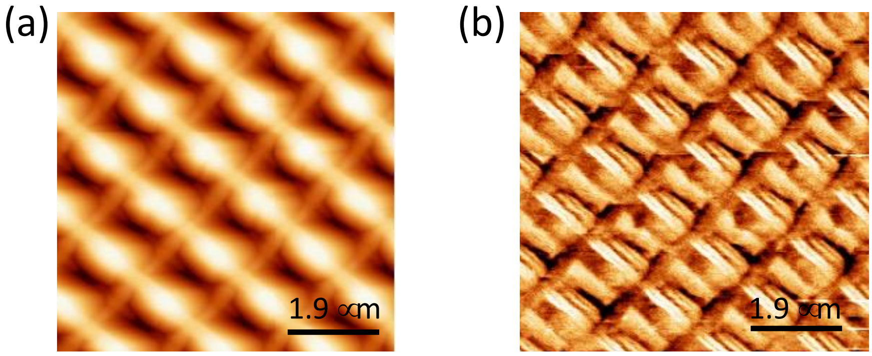

Figure S2: (a) Atomic force microscopy image of representative sample that was subject to Au depositions upon the sidewalls, and Py from normal incidence. (b) Corresponding magnetic force microscopy image.

S2. Bias Field Dependent Spin-Wave Frequency:

Despite the complex structure of the 3D-ASI studied in this work, we attempted to fit the bias field dependent frequency of the dominant spin-wave mode of the studied structure with Kittel

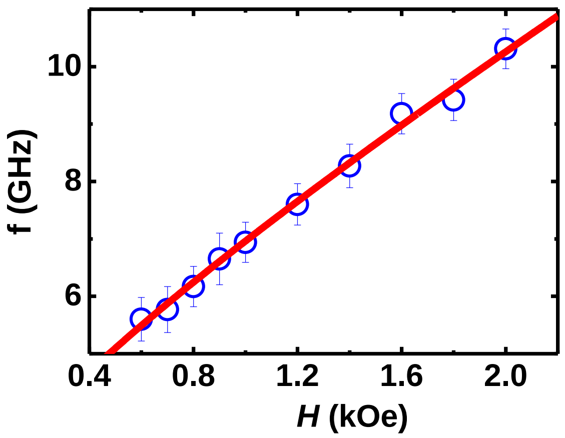

Figure S3: Experimental data of bias field dependent spin-wave frequency (open symbols) fitted with Kittel formula of Eq. S1 (solid line).

formula [3]. Considering the applied bias magnetic field along x direction and zero magneto-crystalline anisotropy in our sample we write the Kittel formula as:

$$f = \frac{\gamma}{2\pi}[(H + H_{d1})(H + H_{d2})]^{\frac{1}{2}} \qquad [S1]$$

Here, γ is gyromagnetic ratio, H is external applied magnetic field, $H_{d1} = (N_z - N_x)M_s$ and $H_{d2} = (N_y - N_x)M_s$ are the effective demagnetizing fields originating from the shape of the studied structure, and M_s is saturation magnetization. Here, N_x, N_y and N_z are the demagnetizing factors along x, y and z direction, respectively. The fitted curve is shown in Fig. S3. The experimental data was replotted from Fig. 3(a) of the article. From the fit we find the values of demagnetizing factor as N_x = 2.54, N_z = 2.82 and N_y = 7.2 considering the values of γ = 17.6 MHz/Oe and M_s = 860 emu/cc. The extracted values of demagnetizing factors suggest that our sample has a relatively high demagnetization contribution along out-of-plane direction (along y, shown in Fig. S5).

S3. Static Magnetization Configuration of the Sample:

The simulated magnetization configuration (m_x component) of 3D-ASI sample at different bias field is shown in Fig. S4. The magnetization profiles show saturated state in applied field regime from 0.6 kOe to 2 kOe. All the measurements were done in the saturated state of the sample.

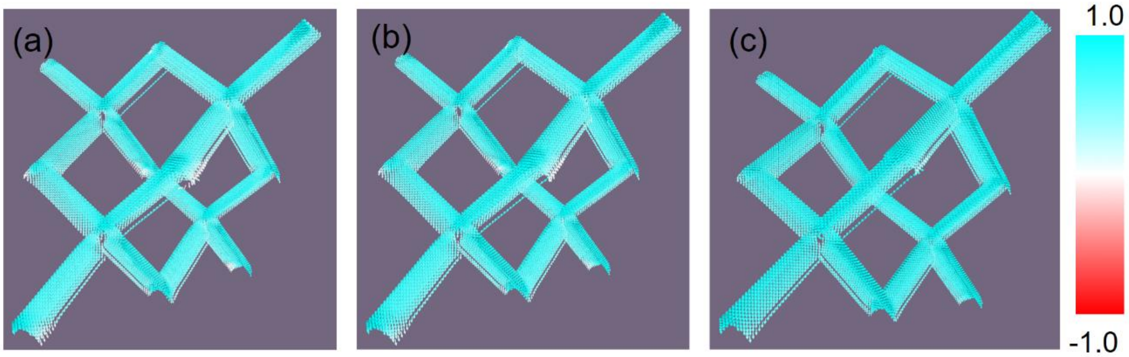

Figure S4: Static magnetization configurations (m_x component) of the sample at bias magnetic field (H) of (a) 0.6 kOe, (b) 1.0 kOe and (c) 2.0 kOe are shown.

The equilibrium state (static) magnetization configuration of two orthogonal components (m_y and m_z) at H = 1.6 kOe applied along x direction (m_x is already shown in Fig. 4(c) of main article) is presented in Fig. S5. Two orthogonal components show prominent demagnetized regions in the magnetization configuration. A distinct difference in the spin structures in the out-of-plane component (m_y component), including the demagnetization regions between intra- and inter-nanowires branches is observed. The out-of-plane configuration (m_y) shows more dominant demagnetized state compared to the in-plane configuration (m_z) which also validates the extracted results of higher demagnetizing factor (N_y) along the out-of-plane direction as presented in S2.

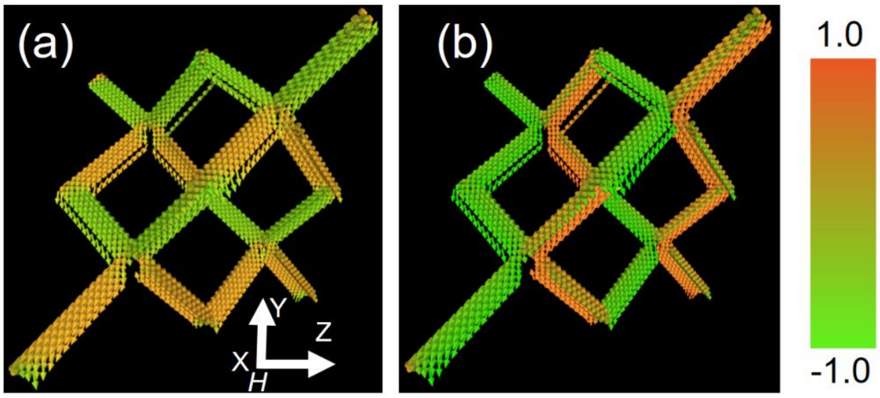

Figure S5: Static magnetization configuration of (a) m_z component and (b) m_y component of the 3D-ASI sample at H = 1.6 kOe applied in the plane of the sample (along x direction).

S4. Collective Nature of Spin-Wave Modes:

In order to check the collective nature of the SW modes, we have simulated the SW spectra at H = 1.6 kOe for a single nanowire leg, one tetrapod element and a unit cell of 3D-ASI structure as shown in Fig. S6. The simulated SW spectra of all three-test structures show distinct behaviours in SW frequency peaks and a gradual evolution from a single nanowire to 3D-ASI structure. These test simulations reassure the collective SW dynamics of the 3D-ASI structure observed in our experiment and simulation.

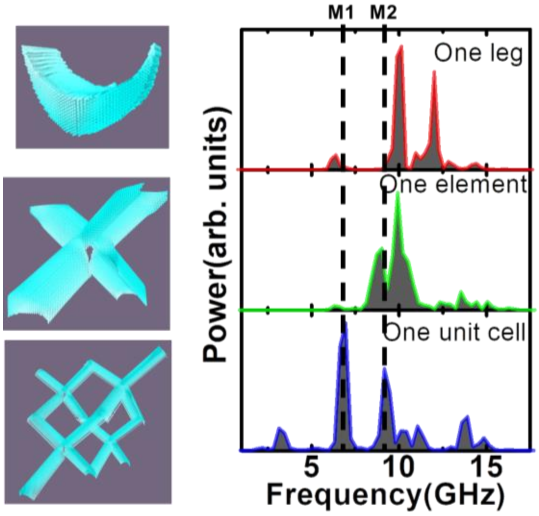

Figure S6: Simulated spin-wave spectra for one leg, one element and one unit cell of 3D-ASI at H = 1.6 kOe are shown. The positions of M1 and M2 are marked by black dashed lines. Representative simulated structures are shown on the left panels.

S5. Spin-Wave Mode Profile Calculation Procedure:

In the dynamic simulations MuMax3 software generates ".ovf" files with spatial distribution of magnetization ($M(t,x,y,z)$) at a particular given time which contains the information of superposed multiple resonant modes. The calculation of power and phase profiles of the multiple resonant modes requires further processing of the data. In order to extract the power and phase profiles of the multiple resonant modes we analysed the ".ovf" files using our home built post processing code DOTMAG [4,5]. To calculate the power and phase profiles in the frequency domain, it takes the fast Fourier transformation (FFT) of time-domain data along a plane of the sample by keeping one of the coordinates fixed (either x or y or z). If we fix $z = z_1$ then the FFT is taken along the x-y plane: $\widetilde{M}^{Z_1}(f,x,y) = FFT\left(M^{z_1}(t,x,y)\right)$. Then the power and phase are calculated for a resonant mode of particular frequency $f = f_1$ which can be written as:

Power: $P^{z_1 f_1}(x,y) = 20log_{10}\left|\widetilde{M}^{z_1}(f_1,x,y)\right|$ and

Phase: $\phi^{z_1 f_1}(x,y) = \mathrm{atan2}\left(Im\left(\widetilde{M}^{z_1}(f_1,x,y)\right), Re\left(\widetilde{M}^{z_1}(f_1,x,y)\right)\right)$

S6. Calculated Spin-Wave Mode Profiles of Additional Modes Observed Only in the Simulation:

In the simulated SW spectra, we have observed some additional modes, which were either not resolved or detected due to limitations in resolution and/or detection sensitivity. However, these modes are fundamentally important, as they are characteristic modes of this structure.

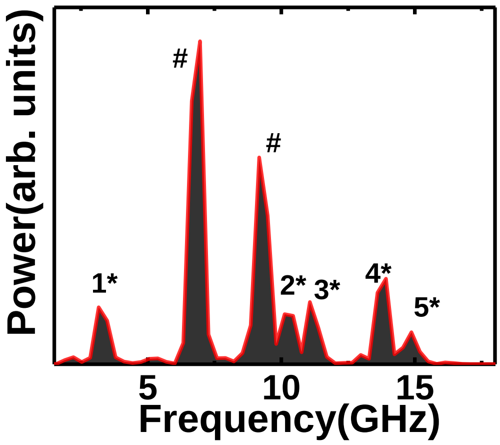

Figure S7: Simulated spin-wave spectra of the 3D-ASI sample at H = 1.6 kOe. The experimentally observed modes are marked by '#' while the additional modes observed explicitly in simulation are numbered as 'digit*' from lower to higher frequencies.

Hence, we have simulated the profiles of these modes too for completeness of study. These modes are numbered from lower to higher frequencies (Fig. S7). Figure S7 is repeated from Fig. 3 (b) of the article for ready correlation with the simulated mode profiles. The SW mode profiles are presented in Fig. S8. Here, M1* shows quantized nature with quantization number n = 11 (Fig. S8(a)) and 11 (Fig. S8(b)). The cross-sectional view shows that it also forms quantized mode along the nanowire cross section (Fig. S8(a)) and at the junction (Fig. S8(c)) with n' = 4 and 3, respectively. Furthermore, M2* and M3* also show quantized behaviour with power distributed over the entire 3D-ASI structure (Fig. S8(a) and (b)). The quantization number is found to be n = 5 and 7 for M2* and n = 7 and 6 for M3*, as shown in Fig. S8(a) and (b), respectively. The cross-sectional view shows the quantized nature of the modes with n' = 2, 2 and 4, 2 for M2* and M3*, respectively, at the cross section (Fig. S8(a)) and the

junction(Fig. S8(c)). The two higher frequency modes also show the quantized nature along the lateral directions of the connected nanowires (Fig. S8(d) and (e)) with n = 12 and 12 for M4* and n = 11 and 15 for M5*. The cross-sectional view of the nanowire (Fig. S8(d)) shows quantized nature of M4* with quantization number n' = 6, while M5* shows a uniform mode. The cross-sectional view at the junction (Fig. S8(f)) shows the quantized nature with n' = 3 and

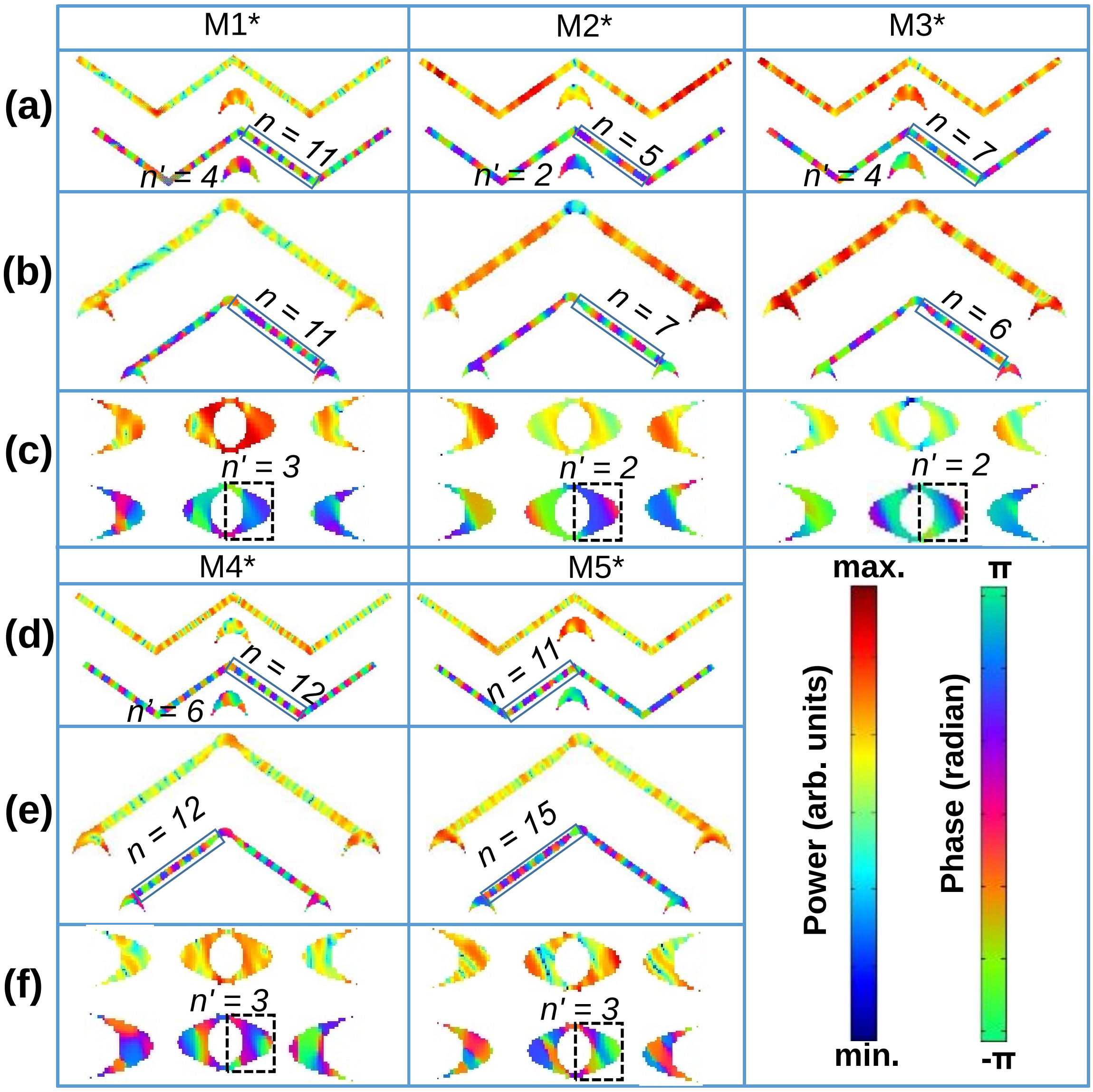

Figure S8: Spin-wave mode profiles calculated at different points of 3D-ASI structure at H = 1.6 kOe by taking slice along (a), (d) x´-y´ plane at point 1, (b), (e) y´-z´ plane at point 2 and (c), (f) x´-z´ plane at point 1. The mode profiles of M1, M2 and M3 are shown in (a), (b) and (c), while (d), (e) and (f) show the mode profiles of M4* and M5*. The x´-y-´z´ coordinate and positions (1 and 2) are presented in Fig. 5(a) of the main article. The power profiles are shown in upper part and corresponding phase profiles in lower part of each panel. The color bars are presented at bottom right corner.

3 for M4* and M5*, respectively. The analyses of SW mode profiles reveal primarily quantized nature of these observed additional modes in simulated spectra with significant amount of power distributed over the entire 3D-ASI structures. The phase profiles reveal the 3D nature of these modes with different quantization number and nature along the different lateral directions and cross sections of the connected nanowires of the 3D-ASI structure.

S7. Spin-Wave Mode Profiles of Adjacent Frequencies:

Due to its finite width each SW peak profile is formed of a range of frequency points instead of a single frequency line. Although we generally present the SW power and phase maps of the

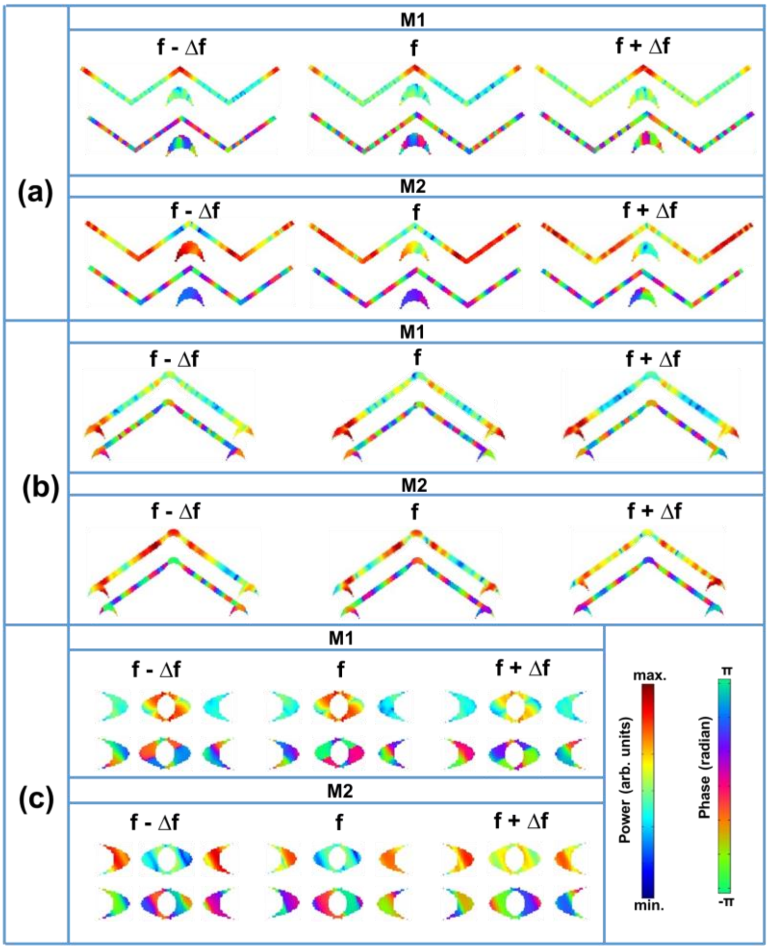

Figure S9: Spin-wave mode profiles calculated at different frequency slices of 3D-ASI structure at H = 1.6 kOe by taking slice along (a) x´-y´ plane at point 1, (b) y´-z´ plane at point 2 and (c) x´-z´ plane at point 1. The x´y´z´ coordinate and positions (1 and 2) are presented in Fig. 5(a) of the article. The power profiles are shown in upper panel and corresponding phase profiles in lower panel of each section. The color bars are presented at bottom right corner.

peak frequency (frequency corresponding to the highest intensity of the SW peak profile), the adjacent frequency slices also carry important information about the mode. Here, we have presented SW power and phase maps of the two adjacent frequency slices (Δf = 0.2 GHz) in addition to the central peak frequency in Fig. S9. The mode profiles of adjacent frequency slices are similar to the central peak frequency, while in some cases the power and phase map resolve the nodal planes of the quantized modes better. We believe this will also clarify the correspondence between power and phase profiles of individual modes. For details of mode profile calculation, we refer to Fig. 5 and its discussion in the article.

Supplementary References:

[1] A. May, M. Hunt, A. Van Den Berg, A. Hejazi, and S. Ladak, "Realisation of a frustrated 3D magnetic nanowire lattice," Commun. Phys. **2**, 13 (2019).

[2] A. May, M. Saccone, A. van den Berg, J. Askey, M. Hunt, S. Ladak, "Magnetic Charge Propagation upon a 3D Artificial Spin-ice". arXiv:2007.07618.

[3] C. Kittel, "On the Theory of Ferromagnetic Resonance Absorption," Phys. Rev. **73**, 155 (1948).

[4] D. Kumar, O. Dmytriiev, S. Ponraj, and A. Barman, "Numerical calculation of spin wave dispersions in magnetic nanostructures," J. Phys. D: Appl. Phys. **45**, 015001 (2011).

[5] G. Venkat, D. Kumar, M. Franchin, O. Dmytriiev, M. Mruczkiewicz, H. Fangohr, A. Barman, M. Krawczyk, and A. Prabhakar, "Proposal for a Standard Micromagnetic Problem: Spin Wave Dispersion in a Magnonic Waveguide," IEEE Trans. Magn. **49**, 524 (2013).
